# Model Based Identification of Linezolid Exposure–toxicity Thresholds in Hospitalized Patients

**DOI:** 10.3389/fphar.2021.732503

**Published:** 2021-10-05

**Authors:** Jie Fang, Xiao-Shan Zhang, Chun-Hong Zhang, Zi-Ye Zhou, Lu Han, Ye-Xuan Wang, Xiao-Shuang He, Xiao-Lan Bian, Guan-Yang Lin, Zheng Jiao, Ying Dai, Xu-Ben Yu, Jing-Ye Pan

**Affiliations:** ^1^ Department of Pharmacy, School of Medicine, Ruijin Hospital, Shanghai Jiaotong University, Shanghai, China; ^2^ Department of Pharmacy, Wenzhou Medical University, Wenzhou, China; ^3^ Department of Pharmacy, The First Affiliated Hospital of Wenzhou Medical University, Wenzhou, China; ^4^ Department of Pharmacy, Shanghai Chest Hospital, Shanghai Jiao Tong University, Shanghai, China; ^5^ Intensive Care Unit, The First Affiliated Hospital of Wenzhou Medical University, Wenzhou, China

**Keywords:** threshold, therapeutic drug monitoring, population pharmacokinetics, linezolid, toxicicity

## Abstract

Evidence supports linezolid therapeutic drug monitoring as the exposure–response relationship has been identified for toxicity among patients receiving linezolid, but the data to establish the upper limit are limited and the published toxicity thresholds range widely. The purpose of this study was to determine the linezolid exposure–toxicity thresholds to improve the safety of linezolid. This is a multicenter retrospective study of adult patients treated with linezolid from 2018 to 2019. The population pharmacokinetic model of linezolid was established based on 270 plasma concentrations in 152 patients, which showed creatinine clearance and white cell count are covariates affecting the clearance of linezolid, and serum albumin is the covariate affecting the volume of distribution. Classification and regression tree analysis was used to determine the linezolid exposure thresholds associated with an increased probability of toxicity. Among 141 patients included for toxicity analysis, the rate of occurring toxicity was significantly higher among patients with an AUC_0-24, d1_ ≥163 mg h/L, AUC_0-24, d2_ ≥207 mg h/L, AUC_0-24, ss_ ≥210 mg h/L, and C_min,d2_ ≥6.9 mg/L, C_min,ss_ ≥6.9 mg/L, while no threshold was discovered for C_min, d1_. Those exposure thresholds and duration of linezolid treatment were independently associated with linezolid-related toxicity in the logistic regression analyses. In addition, the predictive performance of the AUC_0-24_ and C_min_ thresholds at day 2 and steady state were close. Considering that the AUC estimation is cumbersome, C_min_ threshold at 48 h and steady state with a value of ≥6.9 mg/L is recommended to improve safety, especially for patients with renal insufficiency and patients with low serum albumin.

## Introduction

Linezolid, the first oxazolidinone agent, is marketed against Gram-positive bacteria, including those resistant to beta-lactams and glycopeptides such as methicillin-resistant *Staphylococcus aureus* and vancomycin-resistant *Enterococcus faecium* (Vardakas et al., 2007; Metaxas and Falagas, 2009; Mendes et al., 2014). The current FDA-approved label recommends that dose adjustment is unnecessary for patients at any stage of renal dysfunction, including hemodialysis, even though the clearance (CL) of linezolid was found to increase by 50% during hemodialysis (Brier et al., 2003). However, linezolid concentrations are significantly higher in patients with renal impairment than those in patients without (Lin et al., 2006; Matsumoto et al., 2009; Tsuji et al., 2011; Pea et al., 2012; Tsuji et al., 2013; Cossu et al., 2014; Matsumoto et al., 2014). Patients with renal insufficiency are more likely to experience linezolid-related adverse events, mainly hematological toxicity (Wu et al., 2006; Tsuji et al., 2011; Cattaneo et al., 2013; Tsuji et al., 2013; Matsumoto et al., 2014). Moreover, patients undergoing peritoneal dialysis (Roger et al., 2016) or hemodialysis are more likely to experience linezolid-related hematological and metabolic complications (Tsuji et al., 2008).

Thrombocytopenia and anemia are the main adverse effects of linezolid. Besides, linezolid is also associated with metabolic toxicity (lactic acidosis), gastrointestinal disorders, leukopenia, and neurological toxicity (peripheral neuropathy). Long-term use of linezolid increases the possibility of thrombocytopenia and anemia, and duration of linezolid treatment of more than 11–15 days was significantly associated with the development of thrombocytopenia (Matsumoto et al., 2014; Ichie et al., 2015). In addition, a clear exposure–response relationship has been identified for thrombocytopenia among patients treated with linezolid (Cattaneo et al., 2016). The first study on therapeutic drug monitoring (TDM) of linezolid was published by Mastsumoto et al. (2010) who developed a logit model equation identifying a linezolid concentration of 22.1 mg/L as the upper therapeutic threshold for inducing thrombocytopenia (Hiraki et al., 2012). However, this proposed therapeutic target had been challenged by Pea et al. who identified much lower thresholds at a trough concentration of 6.5 mg/L and/or AUC_0-24_ of 280 mg h/L (Pea et al., 2012). More recently, Matsumoto et al. (Matsumoto et al., 2014) suggested the linezolid trough thresholds <8.2 mg/L as the upper limit to minimize the risk of linezolid-induced thrombocytopenia. In further support of this target, a recently published pharmacokinetic (PK)/toxicodynamic model has shown that a concentration of linezolid >8.1 mg/L inhibited 50% of the platelet precursor cell synthesis (Boak et al., 2014). The suggested TDM target of linezolid from different studies is summarized in Table 1. However, due to the C_min_ estimation methods varied across those studies, the thresholds ranged widely from 6.5 mg/L to 22.1 mg/L. In addition, almost all of the studies defined the upper therapeutic threshold only based on the development of thrombocytopenia, the risk of anemia, and lactic acidosis, which also have a high rate of toxicity in the linezolid treatment, have not been investigated in defining the TDM target. What is more, except Pea et al. who defined the threshold by AUC_0-24_, other studies defined the safety target by trough concentration. The purpose of this analysis is to derive linezolid exposure–toxicity thresholds in hospitalized patients and to compare the threshold defined by AUC and trough concentration at different time periods during the treatment.

**TABLE 1:** Summary of studies dealing with the safety TDM target of linezolid.

Reference	Study design	Patient population	n	Suggested dosing	Suggested safety target	Method for defining the target
Nukui et al. (2013)	Prospective, observational	Patients given LZD for any reason	41	600 mg q12 h	C_min_< 8.1 mg/L (for hematological toxicity)	PK/TD model (inhibited the synthesis of platelet precursor cells by 50%)
Cattaneo et al. (2013)	Prospective, observational	Patients given LZD for any reason	50	600 mg q12 h	C_min_< 9.0 mg/L (for hematological toxicity)	Logistic regression model
Hiraki et al. (2012)	Retrospective	Patients with pneumonia	8	Nm	C_min_< 22.1 mg/L (for thrombocytopenia)	Logit model
Matsumoto et al. (2014)	Retrospective	Patients given LZD for any reason	44	Initial daily dose (mg/day) = CL × AUC24= (0.0258 × CLCr+ 2.03) × (18.2 × Cmin+ 134.4)	C_min_ < 8.2 mg/L (for thrombocytopenia)	Logistic regression model
Tsuji et al. (2017)	Prospective, observational	Patients given LZD for any reason	30	High-risk patients:600 mg/day	C_min_< 7.5 mg/L (for thrombocytopenia)	ROC curve analysis
Pea et al. (2012)	Retrospective	Patients given LZD for any reason	45	600 mg q12 h	AUC_0-24_ <280 mg h/L or C_min_ <6.5 mg/L (for thrombocytopenia)	Logistic regression model

nm, not mentioned; LZD, linezolid.

## Materials and Methods

### Patients and Ethics

This was a multicenter, retrospective, and observational study of hospitalized adult patients receiving linezolid for confirmed or suspected multiresistant Gram-positive bacterial infections from January 2018 to December 2019 at the First Affiliated Hospital of Wenzhou Medical University and Ruijin Hospital, Shanghai Jiaotong University School of Medicine. The inclusion criteria were as follows: 1) patients ≥18 years with confirmed or suspected multiresistant Gram-positive bacterial infection; 2) patients who received intravenous and/or oral linezolid for at least 10 days; and 3) at least one steady-state concentration of linezolid was collected. The exclusion criteria were as follows: 1) patients who received renal replacement therapy or extracorporeal membrane oxygenation and 2) patients who died within 24 h after being treated with linezolid. The patients who were included for toxicity analysis were further excluded if 1) baseline PLT <75 × 10^9^ cells//L, 2) baseline hemoglobin <6.8 g/dl for males or 6 g/dl for female individuals, 3) baseline absolute neutrophil count <500 cells/μL, and 4) baseline total bilirubin > 5-times the upper limit of normal. The baseline was defined as the initiation of linezolid therapy.

Patient data including demographics, comorbidities, medication therapy, laboratory values, physiologic parameters, and indication for linezolid therapy were obtained from the electronic medical record. Renal function was assessed by serum creatinine and creatinine clearance (CrCL) estimated by the Cockcroft–Gault formula. Data organization and visualization were performed using R (version 3.6.0) and R Studio (version 1.2.1335).

The study was designed in accordance with legal requirements and the Declaration of Helsinki and was approved by the Ethical Committee of the First Affiliated Hospital of Wenzhou Medical University, China ([2021]034) and the Ethical Committee of Ruijin Hospital (KY2020-68). The study has been registered at the Chinese Clinical Trial Registry (ChiCTR2100047882). The informed consent was passed by the ethics committee in clinical research.

### Pharmacokinetic Sampling

Routine clinical TDM data of patients treated with linezolid were retrospectively obtained from a database maintained at the Department of Pharmacy. The decision to administer linezolid and its dosing regimens (dose amount, dosing interval, duration of intravenous administration, and duration of therapy) was made by the attending physician. An opportunistic sampling strategy was performed when a steady-state concentration (attained after at least five continuous doses) of linezolid has been achieved. Dates and the exact time of linezolid treatment and TDM sampling were able to be indexed.

Plasma samples were separated by centrifugation for 5 min at 15,000 rpm immediately after sampling and stored at −80°C. Samples were determined within 24 h after sampling. The quantification of plasma concentration of linezolid was performed using a validated high-performance liquid chromatography–tandem mass spectrometry assay (Dai et al., 2021). The intra- and inter-day assay coefficients of variation were <10%, and the lower limit of quantification was 0.1 mg/L.

### Toxicity Analysis of Linezolid

Toxicity was defined as follows: 1) thrombocytopenia: platelet count <125 × 10^9^ cells/L and a decrease of platelet count ≥25% in comparison with baseline levels; 2) anemia: a reduction of ≥25% of hemoglobin level compared to the baseline level; 3) leukopenia, white blood cell (WBC) count <4.0 × 10^9^ cells/L; and 4) hyperlactacidemia: serum pH < 7.35 and serum lactate >4 mmol/L (Dai et al., 2021). The baseline levels were defined at the initiation of linezolid therapy.

### Population Pharmacokinetic Modeling of Linezolid

PopPK analysis was performed using non-linear mixed-effects modeling program NONMEM (version 7.4, Icon Development Solutions, Ellicott City, MD, United States) and Pirana (version 2.9.7). R (version 3.6.0) and Xpose (version 4.3.2) software packages were applied to generate diagnostic plots. The first-order conditional estimation method with inter- and intra-subject variability was used throughout the model development procedure.

One- and two-compartment structural models with first-order elimination were explored for the linezolid plasma concentration–time profiles. Between-subject variability (BSV) was modeled using exponential function. Residual variability was assessed using additive, proportional, and combined (additive plus proportional) error models. The base model was selected based on the visual inspection of diagnostic plots and various goodness-of-fit criteria, including precision and plausibility of parameter estimation, improvement of the objective function value (OFV), and the Akaike Information Criteria and Bayesian information criterion.

Exploratory analysis was performed before covariate modeling to examine the distribution of covariates in the population and the correlation between covariates of interest. Primary covariates included gender, age, height, body weight, white blood cell count, hemoglobin, platelet count, total bilirubin, albumin (ALB), alanine aminotransferase, aspartate aminotransferase, serum creatinine, and CrCL. Relationships between individual empirical Bayesian estimates of PK parameters and patient covariates were examined visually. Covariates were included using a stepwise forward selection process, with a threshold decrease in the OFV of 3.84 [*p* < 0.05, 1 degree of freedom (df)] until no further decrease in OFV was observed. All the significant covariates were then incorporated into the basic model to construct a full model. In backward elimination, the covariate was retained in the final model with a threshold increase in the OFV of 6.63 (*p* < 0.01, 1 df); otherwise, it was eliminated from the model. The additional criterion for retaining the covariate in the final model was a decrease in the unexplained BSV and an increase in PK parameter estimate precision.

Goodness-of-fit plots, non-parametric bootstrap, and visual predictive check were performed to evaluate the final model and parameter estimates. Goodness-of-fit plots include observed concentrations versus individual prediction, observed concentrations versus population prediction, conditional weighted residuals versus population prediction, and conditional weighted residuals versus time after the last dose. A non-parametric bootstrap procedure was conducted to assess the performance and stability of the final model. Random sampling with replacement was utilized to generate 1,000 replicate datasets using the individual as the sampling unit. The median and 95% confidence intervals of the resulting parameters were calculated and compared with the final parameter estimates obtained using NONMEM program. To evaluate the predictive performance, the statistics of the observed and simulated time–concentration profiles were compared using prediction- and variability-corrected visual predictive check. The dataset was simulated 1,000 times using the SIMULATION block in NONMEM for prediction- and variability-corrected visual predictive check. The 95% CI for the 5^th^, 50^th^, and 95^th^ percentiles of the simulated concentrations were calculated, plotted against time after the last dose, and compared with the observed concentrations.

### Statistical Analyses

Statistical analyses were performed using SPSS version 21.0 (IBM Corp). All study variables were summarized by descriptive statistics. The PK parameters such as the AUC_0-24_ and C_min_ at day 1 (AUC_0-24, d1_ and C_min, d1_), day 2 (AUC_0-24, d2_ and C_min, d2_), and steady state (AUC_0-24, ss_ and C_min, ss_) of each patient were predicted via the maximum a posteriori probability (MAP) Bayesian function of NONMEM using the final PK model as the Bayesian prior. The Kolmogorov–Smirnov test was performed to assess whether the data were normally or non-normally distributed. The relationship between the linezolid concentration–time profile and linezolid-associated toxicity was examined. Categorical variables were compared by using the chi-squared or Fisher exact test, and the continuous variables were compared using the Student *t* test or Mann–Whitney *U* test, as appropriate, comparing toxicity versus non-toxicity and dichotomous linezolid exposure thresholds.

Thresholds in the distribution of the linezolid exposure variables where the incidence of toxicity was most disproportionate were derived using the classification and regression tree (CART) analysis (Zhang, 1999). The predictive performance of linezolid exposure variables, including the CART-derived and other prior defined exposure thresholds, was evaluated using receiver operating characteristic (ROC) curves along with negative predictive value and positive predictive value (NPV and PPV, respectively). Multivariable logistic analyses were performed to determine the independent association between linezolid exposure variables and toxicity, while adjusting for confounding variables. Each linezolid exposure variable was assessed in a separate logistic regression model. All baseline variables associated with toxicity in the bivariable model analyses at a *p* < 0.1 were included in the explanatory multivariable model at model entry, and variables were excluded from the model using a backward stepwise approach. All statistical tests were two-sided. *p* values < 0.05 were considered statistically significant.

## Results

### Baseline Characteristics of Patients

In total, 152 hospitalized adult patients with 270 plasma concentrations were included in the population PK analysis set; 141 patients were evaluable for hematological toxicity analysis. The demographic data and clinical baseline characteristics of these patients are summarized in Table 2, and the demographic profiles were similar across different analysis sets. The overall toxicity rate, including thrombocytopenia, anemia, leukopenia, and lactic acidosis, in this study was 43.26%. The median daily dose of linezolid selected by the physician was 18.83 mg/kg/d. The median [IQR] time of the sampling was 7.0 [4.0–10.0] days.

**TABLE 2:** Baseline characteristics of patients included for pharmacokinetic analysis set or hematological toxicity analysis set.

Characteristic	Pharmacokinetic analysis set	Toxicity analysis set
Age (years)	65 [14, 99]	66 [16, 99]
Sex		
Male	99 (65.13%)	92 (65.25%)
Female	53 (34.87%)	49 (34.75%)
Height (cm)	167 [160, 170]	167 [160, 170]
Total body weight (kg)	64.26 ± 15.82	63.02 ± 13.71
Toxicity		61 (43.26%)
Thrombocytopenia		50 (35.46%)
Anemia		27 (19.15%)
Lactic acidosis		13 (9.22%)
Leukopenia		8 (6.57%)
Clinical data		
Hemoglobin (g/L)	104.86 ± 21.21	105.45 ± 20.68
Platelet (×10^9^/L)	265.58 ± 122.44	254.16 ± 118.25
White cell count (×10^9^/L)	10.33 [6.95, 15.83]	10.06 [6.77, 15.21]
Total bilirubin (μmol/L)	10 [7, 13]	10 [7, 14]
AST (U/L)	28 [19, 46]	29 [20.5, 50.5]
ALT (U/L)	31 [14.5, 47]	27 [15, 48]
ALB (g/L)	30.09 ± 5.84	29.83 ± 6.00
Serum creatinine (μmol/L)	73 [58.5, 120]	74 [56, 122]
CrCL (ml/min)	76.24 ± 41.03	74.09 ± 40.78

ALB, serum albumin; ALT, alanine transaminase; AST, aspartic transaminase; CrCL, estimated creatinine clearance (CrCL) calculated using the Cockcroft–Gault equation

a Values are No. (%) or median [IQR] or mean ± SD.

### Population Pharmacokinetic Analysis

**FIGURE 1:**
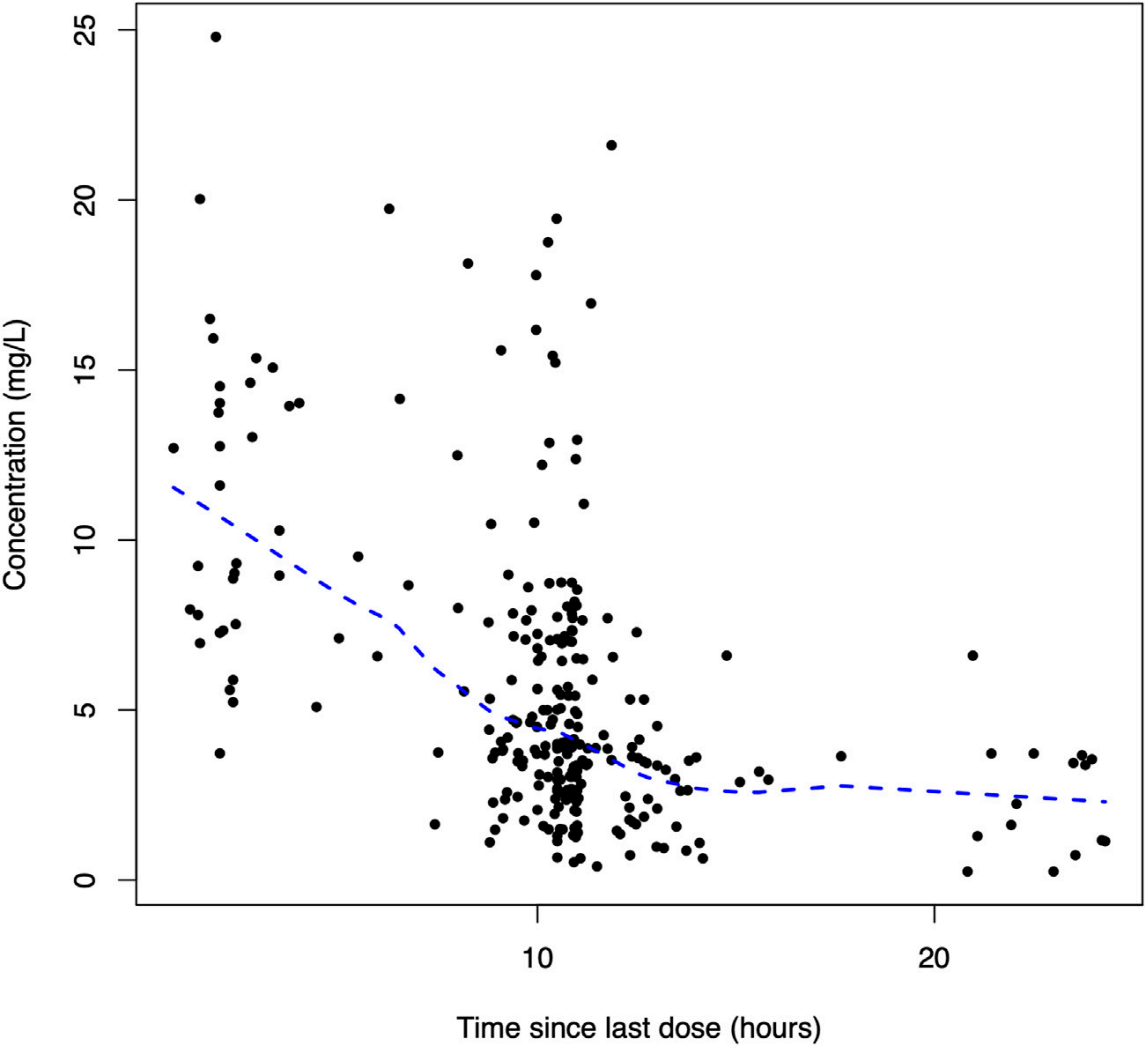
Dose-normalized serum concentration–time profiles of linezolid.

A total of 270 linezolid concentrations from 152 patients with a range of 0.25–24.79 mg/L were obtained for PopPK modeling. The linezolid concentration versus time after the last dose profile is shown in Figure 1.

The PK characteristics of linezolid could be well-illustrated by a 1-compartment model, with linear elimination showing the best fit of the observed concentration–time data based on the reduction in OFV and residual variability compared to the 2-compartment model. BSV was successfully estimated on both CL and V but not the absorption rate constant (Ka) and bioavailability (F) in the base model. The proportional error model was selected to evaluate the residual variability. Parameter estimates and diagnostic plots from the base model are provided in Supplementary Table S1, Figure S1.

The stepwise forward selection and backward elimination process are provided in Supplementary Tables S2, S3. The covariate model building identified CrCL and WBC as covariates of linezolid CL and ALB as a covariate of V. These covariates were not strongly correlated with one another in the population (|r|≤0.4). The final PopPK model is represented as follows:

F=80.6% (1)

$$KA\left(h^{-1}\right)=0.753$$ (2)

$$CL\left(\frac{L}{h}\right)=2.93+2.33\times \frac{CrCL}{73}+0.685\times \frac{WBC}{9.63}$$ (3)

V(L)=97.5−1.42×ALB (4)

where CL is the individual clearance, V is the individual volume of distribution, CrCL is the estimated creatinine clearance, WBC is the white cell count in ×10^9^/L, and ALB is the serum albumin in g/L. The parameter estimates of the final model are displayed in Table 3.

**TABLE 3:** Population pharmacokinetic parameter estimates from the final model.

Parameter	Estimate	RSE (%)	Shrinkage (%)
**Fixed effects**			
TVF	0.731	9	
TVKA[h^−1^]	0.87	23	
TVCL[L/h]	3.07	12	
CrCL on CL	2.37	15	
WBC on CL	0.559	23	
TVV[L]	93.1	15	
ALB on V	−1.33	26	
**Between-subject variability (BSV)**			
BSV_CL [%CV]	32.40%	8	10
BSV_V [%CV]	33.6%	24	18
**Residual variability (RV)**			
Proportional error [%CV]	16.09%	16	14

a BSV calculated as $\sqrt{e^{\omega^{2}}-1}$ ;

TVF, typical value of bioavailability; TVKA, typical value of rate constant (Ka), TVCL, typical value of clearance, TVV, typical value of volume, CrCL, estimated creatinine clearance; WBC, white blood cell count; ALB, albumin; BSV: between-subject variability.

Mean ± SD individual empirical Bayesian estimates of CL and V were 6.36 ± 2.50 L/h and 53.98 ± 14.59 L, respectively, across all patients with F estimated at 0.81 and KA estimated at 0.75 h^−1^ in the population. Furthermore, patients included for toxicity analysis were divided into the group with linezolid-associated toxicity and the group without. The results of the Student *t* test indicated that the estimated CL values were significantly lower in patients with linezolid-associated toxicity than patients who did not experience toxicity (7.08 ± 2.65 L/h vs 5.86 ± 2.23 L/h). But no statistically significant difference in the estimated V value was observed between the two groups (55.88 ± 17.40 L vs. 52.63 ± 12.09 L).

**FIGURE 2:**
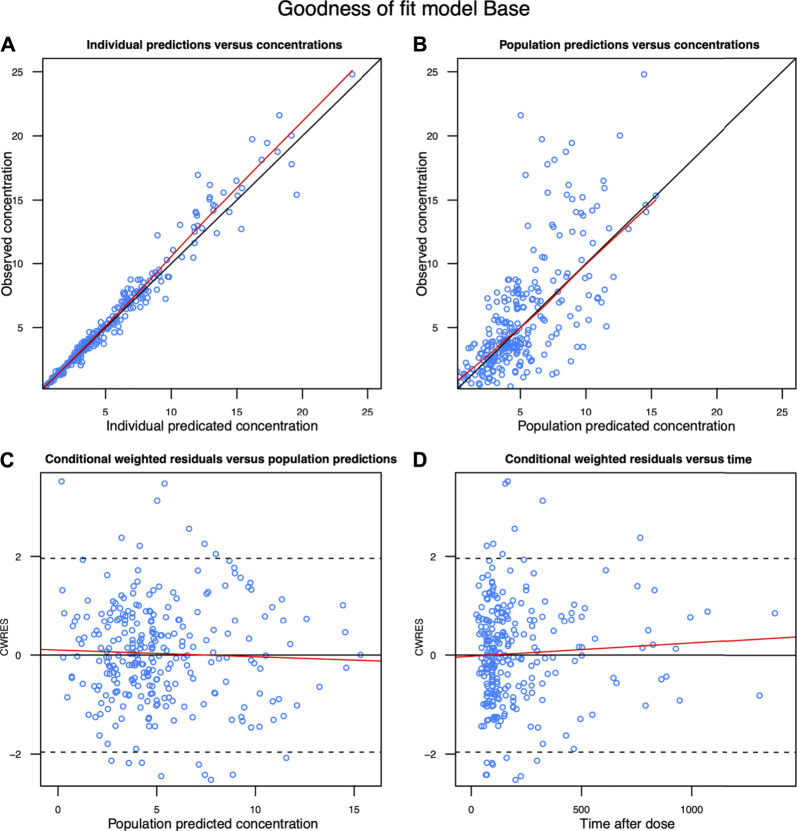
Diagnostic goodness-of-fit plots of the final model. **(A)** Observed concentration (DV) vs. individual predicted concentration (IPRED); **(B)** DV vs population predicted concentration (PRED); **(C)** conditional weighted residuals (CWRES) vs PRED; and **(D)** CWRES vs time. The red lines in the upper panel represent less smooth lines and linear fit lines, respectively.

The diagnostic goodness-of-fit plots of the final model are shown in Figure 2. The scatterplots of population prediction and individual prediction revealed an improvement in the final model compared with that of the basic model. The conditional weighted residuals vs the population prediction of the final model showed a stochastic distribution around zero, and most residuals were within an acceptable range (−2–2). The median with 95% CI parameter estimates obtained from a 1,000-run bootstrap analysis are given in Supplementary Table S4. The parameter estimates of the final PopPK model lie within the 95% CIs resulted from the non-parametric bootstrap procedure, and the biases between the final model estimates and bootstrapped median parameter estimates were < ±10% for all parameters, which demonstrated the good stability of the final model. The prediction- and variability-corrected visual predictive check of concentrations versus time after the last dose reflected a good fit between the observations and simulations (Supplementary Figure S2). Overall, the linezolid PopPK model evaluation results revealed that the final model provided an adequate description of the data and a good prediction of the individual PK parameters.

### Toxicity Analyses

**FIGURE 3:**
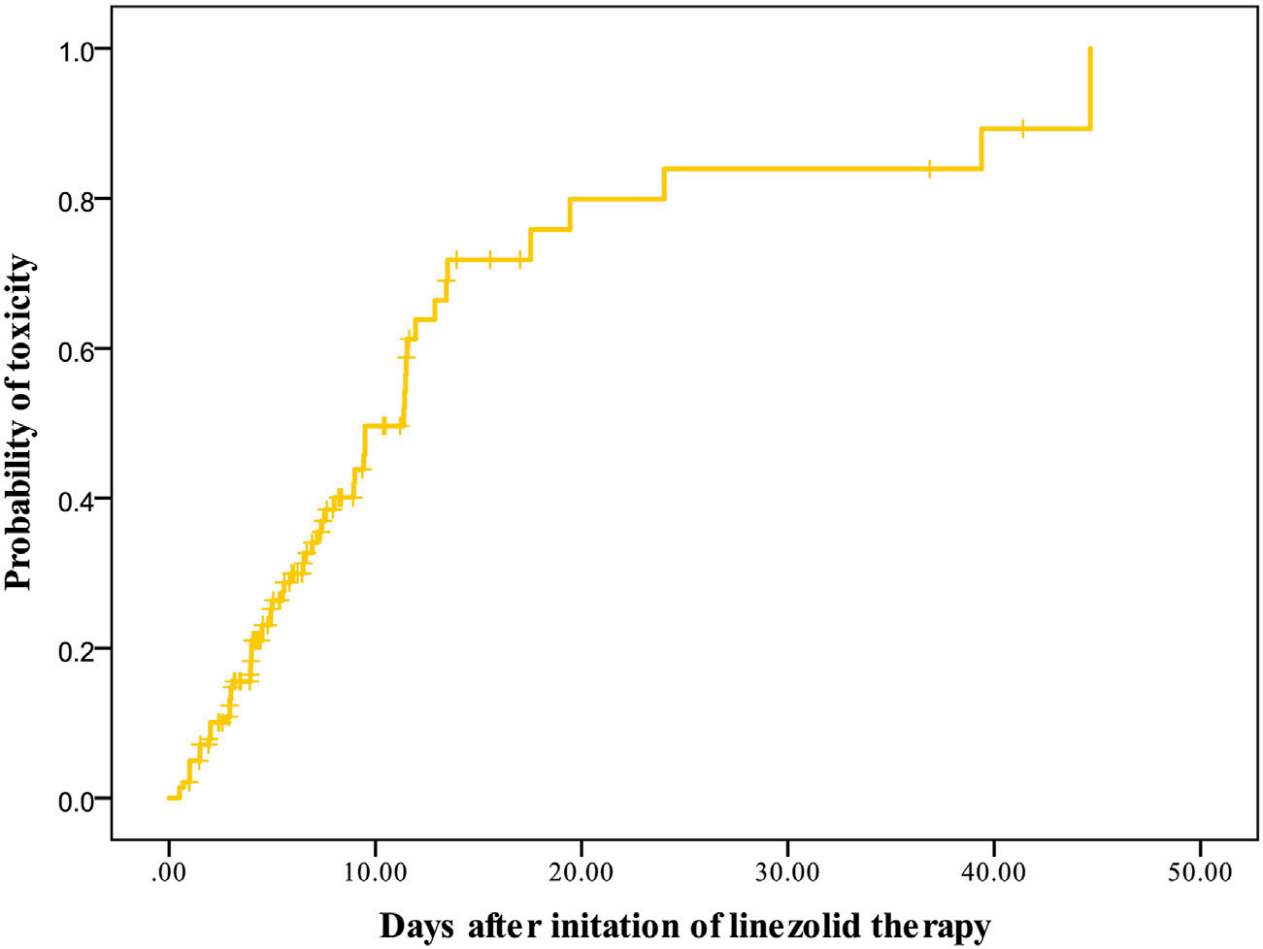
Kaplan–Meier plot showing the time from the initiation of linezolid therapy to the development of toxicity (*n* = 141).

A total of 141 patients met the criteria for toxicity analyses. The incidence rate of thrombocytopenia, anemia, leukopenia, and hyperlactacidemia was 35.46, 19.15, 6.57, and 9.22% respectively. The Kaplan–Meier plot revealed that the median time from the initiation of therapy to the development of linezolid-induced toxicity was 12 days (Figure 3). Bivariate comparisons between patients who experienced and did not experience linezolid-related toxicity are listed in Table 4. Patients who experienced toxicity had significantly lower CrCL and longer treatment duration. In addition, patients with toxicity had significantly higher linezolid exposure quantified by AUC_0-24, d1_, AUC_0-24, d2_, AUC_0-24,ss_, C_min, d1_, and C_min, d2_, or C_min, ss_.

**TABLE 4:** Bivariate comparisons of demographic and clinical characteristics between patients with and without hematological toxicity.

Characteristic	Toxicity		*p* value
	None (*n* = 80)	Yes (*n* = 61)	
Demographics			
Age (years), mean ± SD	62.79 ± 16.10	66.38 ± 14.30	0.104^a^
Male, *n*(%)	51(63.7)	41 (67.2)	0.669^c^
Height (cm), median [IQR]	168 [160, 170.5]	165 [158, 170]	0.177^b^
Weight (kg), mean ± SD	64.89 ± 15.54	60.57 ± 10.47	0.063^a^
Selected comorbidities, *n* (%)			
Renal disease	16 (20.0)	19 (31.1)	0.129^c^
Liver disease	19 (23.8)	12 (19.7)	0.562^c^
Heart failure	7 (8.8)	10(16.4)	0.167^c^
Diabetes	22 (27.5)	20 (32.8)	0.496^c^
Peripheral vascular disease	20 (25.0)	18 (29.5)	0.550^c^
Hypertension	18 (22.5)	21 (29.5)	0.344^c^
Clinical data			
WBC (×10^9^/L), median [IQR]	9.84 [7.02, 15.08]	10.07 [6.22, 15.40]	0.685^b^
TBIL (μmol/L), median [IQR]	10.00 [7.00, 13.00]	10.50 [7.10, 15.10]	0.223^b^
Hemoglobin (g/L), mean ± SD	107.33 ± 22.23	102.95 ± 18.30	0.217^a^
Platelet (×10^9^/L)	274.16 ± 126.20	228.05 ± 102.12	0.088^b^
ALB (g/L), mean ± SD	30.51 ± 5.82	28.91 ± 5.18	0.096^a^
AST (U/L), median [IQR]	28.00 [21.00, 46.50]	30.50 [19.75, 57.25]	0.631^b^
ALT (U/L), median [IQR]	29.00 [16.50, 50.00]	25.00 [13.75, 43.00]	0.224^b^
SCr (μmol/L), median [IQR]	67.50 [53.25, 94]	100.00 [62.50, 147.00]	0.001^b^
CrCL (ml/min), mean ± SD	87.74 ± 44.46	56.05 ± 30.20	0.000^a^
CrCL ≤80 ml/min, *n* (%)	35 (43.8%)	45 (56.3%)	0.000^a^
Linezolid treatment			
Daily dose (mg/kg/d), mean ± SD	19.08 ± 4.16	19.84 ± 4.59	0.305^a^
Duration (days), median [IQR]	4.94 [3.93, 8.30]	7.39 [4.06, 15.43]	0.007^b^
AUC_0-24,d1_ (mg·h/L), mean ± SD	156.58 ± 32.39	206.26 ± 41.74	0.000^a^
C_min,d1_ (mg/L), mean ± SD	5.04 ± 1.89	8.61 ± 2.60	0.000^a^
AUC_0-24,d2_ (mg·h/L), mean ± SD	182.82 ± 48.06	262.17 ± 85.84	0.000^a^
C_min,d2_ (mg/L), median [IQR]	5.34 ± 2.18	9.12 ± 4.09	0.000^a^
AUC_0-24,ss_ (mg·h/L), median [IQR]	194 ± 50.18	285.74 ± 102.59	0.000^a^
C_min,ss_ (mg/L), median [IQR]	5.46 ± 2.23	9.49 ± 4.55	0.000^a^

ALB, albumin; AST, aspartate aminotransferase; ALT, alanine aminotransferase; CrCL, estimated creatinine clearance (CrCL) calculated using the Cockcroft–Gault equation; Hb, hemoglobin; Scr, serum creatinine clearance; TBIL, total bilirubin; and WBC, white blood cell count.

a Student *t.*

b Mann–Whitney U.

c Pearson chi-square test.

In the CART analysis, the rate of occurring toxicity was significantly higher among patients with AUC_0-24, d1_ ≥163 mg h/L, AUC_0-24, d2_ ≥207 mg h/L, AUC_0-24, ss_ ≥210 mg h/L, and C_min,d2_ ≥6.9 mg/L, C_min,ss_ ≥6.9 mg/L, while no threshold was discovered for C_min, d1_. The predictive performance of the CART-derived linezolid thresholds and other candidate thresholds are listed in Table 5. The CART-derived thresholds for AUC_0-24, d1_, AUC_0-24, d2_, AUC_0-24, ss_, C_min, d2_, and C_min, ss_ were the most predictive in receiver operating characteristic (ROC) curve analysis. Compared with other thresholds, the AUC_0-24, d1_ threshold of ≥163 mg h/L had lower predictive value. In addition, it was noted that the predictive performance of AUC_0-24_ and C_min_ at day 2 was close to that at the steady state.

**TABLE 5:** Predictive performance of CART-derived and other candidate toxicity thresholds.^a^

Thresholds	Sensitivity (%)	NPV (%)	Specificity (%)	PPV (%)	Area under ROC curve (95% CI)
AUC_0-24, d1_ (mg·h/L)					
≥150	96.72	94.44	42.50	56.19	0.696 (0.610–0.782)
≥160	95.08	93.75	56.25	62.37	0.757 (0.677–0.836)
≥163	93.44	92.31	60.00	64.04	0.767 (0.688–0.846)
≥170	83.61	84.13	66.25	65.38	0.749 (0.667–0.832)
≥180	73.77	69.23	75	69.23	0.744 (0.660–0.828)
AUC_0-24, d2_ (mg·h/L)					
≥190	91.80	90.00	56.25	61.54	0.740 (0.658–0.822)
≥200	88.52	88.14	65.00	65.85	0.768 (0.688–0.847)
≥207	88.52	89.23	72.50	71.05	0.805 (0.730–0.880)
≥210	83.61	85.51	73.75	70.83	0.787 (0.709–0.865)
≥220	73.77	79.49	77.50	71.43	0.756 (0.673–0.840)
C_min, d2_ (mg/L)					
≥5	96.72	94.29	41.25	55.66	0.690 (0.604–0.776)
≥6	90.16	89.66	65.00	66.27	0.776 (0.697–0.854)
≥6.9	80.33	84.00	78.75	74.24	0.795 (0.718–0.873)
≥8	78.69	83.12	80.00	75.00	0.793 (0.715–0.872)
≥9	67.21	78.02	88.75	82.00	0.780 (0.698–0.862)
AUC_0-24, ss_ (mg·h/L)					
≥190	91.80	89.80	55.00	60.87	0.734 (0.651–0.817)
≥200	88.52	87.72	62.50	64.29	0.755 (0.674–0.836)
≥210	88.52	89.06	71.25	70.13	0.799 (0.723–0.875)
≥220	77.05	81.33	76.25	71.21	0.766 (0.685–0.848)
≥230	70.49	78.31	81.25	74.14	0.759 (0.675–0.842)
C_min, ss_ (mg/L)					
≥5	96.67	94.29	41.25	55.24	0.690 (0.604–0.776)
≥6	90.00	89.66	65.00	65.85	0.776 (0.697–0.854)
≥6.9	81.97	84.93	77.50	73.53	0.797 (0.720–0.875)
≥8	67.21	78.02	88.75	82.00	0.780 (0.698–0.862)
≥9	59.02	74.49	91.25	83.72	0.751 (0.666–0.837)

a AUC, area under the concentration-time curve; CI, confidence interval; NPV, negative predictive value; PPV, positive predictive value; ROC, receiver operating characteristic.

In the final logistic regression models, linezolid AUC_0-24, d1_ ≥ 163 mg h/L, AUC_0-24, d2_ ≥ 207 mg h/L, AUC_0-24,ss_ ≥ 210 mg h/L, C_min,d2_ ≥ 6.9 mg/L, and C_min,ss_ ≥ 6.9 mg/L were still independently associated with linezolid-related toxicity (Table 6). In addition, the treatment duration of linezolid was also included as another independent risk factor in these final models. It was noted that CrCL was included in the final model when the exposure variable was AUC_0-24, d1_ ≥163 mg h/L.

**TABLE 6:** Logistic regression analysis of the risk factors for linezolid-related toxicity.

Variable	Odds ratio (95% CI)		
	Unadjusted	Adjusted	*p* value
AUC_0-24, d1_			
AUC_0-24, d1_≥163 mg h/L	21.375 (7.058–64.735)	13.591 (4.084–45.232)	0.000
Duration	1.059 (1.014–1.104)	1.068 (1.015–1.125)	0.012
CrCL	0.977 (0.966–0.988)	0.983 (0.969–0.996)	0.013
AUC_0-24, d2_			
AUC_0-24, d2_≥207 mg h/L	20.338 (8.042–51.431)	21.521 (7.874–58.819)	0.000
Duration	1.059 (1.014–1.104)	1.067 (1.018–1.118)	0.007
C_min,d2_			
C_min,d2_≥6.9 mg/L	15.132 (6.612–34.631)	16.102 (6.616–38.189)	0.000
Duration	1.059 (1.014–1.104)	1.070 (1.021–1.122)	0.005
AUC_0-24,ss_			
AUC_0-24,ss_≥210 mg h/L	19.118 (7.586–48.179)	20.621 (7.527–56.498)	0.000
Duration	1.059 (1.014–1.104)	1.068 (1.019–1.120)	0.006
C_min,ss_			
C_min,ss_≥6.9 mg/L	14.769 (6.492–33.600)	15.315 (6.350–36.936)	0.000
Duration	1.059 (1.014–1.104)	1.061 (1.013–1.111)	0.012

## Discussion

Over the last couple of years, the interest on TDM of linezolid has consistently increased. This is the largest retrospective study sought to derive linezolid exposure-toxicity thresholds with linezolid-related adverse events in 141 patients and compare thresholds based on the AUC and C_min_ at initial dose and steady state.

First, we constructed a population pharmacokinetic model, which showed that the CrCL and WBC were significant covariates affecting the clearance of linezolid. According to the drug label inserts, it acknowledges that the main metabolites of linezolid may accumulate in patients with renal insufficiency; however, the clinical significance of this accumulation has not been determined. Consequently, no linezolid dose adjustments are required in renal insufficiency patients. Registrational studies identified 600 mg every 12 h as a “one-size-fits-all” dose of linezolid to be administered to patients older than 17 years for the treatment of Gram-positive bacterial infections. However, large inter-individual variability in linezolid PK has been reported subsequently in a wider range of patients (Di Paolo et al., 2010; Dryden, 2011). In fact, patients with renal insufficiency undergoing renal replacement therapy, with low body weight, and the elderly are at the high risk to be overdosed with linezolid if treated with conventional doses, ultimately resulting in the development of severe linezolid-related adverse events (Tsuji et al., 2011; Cattaneo et al., 2013; Tsuji et al., 2013; Matsumoto et al., 2014; Roger et al., 2016; Tsuji et al., 2017). In this study, the patients experienced linezolid-related toxicity have significantly lower CrCL than those not experienced, and the proportion of patients with a CrCL <80 ml/min was significantly different between the two groups. It was consistent with the studies (Tsuji et al., 2008; Matsumoto et al., 2010; Tsuji et al., 2011; Tsuji et al., 2013; Matsumoto et al., 2014; Tsuji et al., 2017) which documented that a patient with renal dysfunction tends to accumulate linezolid and experience more frequent linezolid-related adverse events. In addition, WBC was included as another covariate on the clearance of linezolid. It might be that patients with high white blood cell counts suffer from severe infections, which could increase the individual metabolic rate, resulting in lower plasma concentration of linezolid. Serum albumin was included as the covariate in the volume of distribution in our final pharmacokinetic model. In clinic, critically ill patients are often suffering low serum albumin, accompanied with increased capillary permeability. This capillary leakage results in fluid shifts from the intravascular compartment to the interstitial space. Considering that linezolid has high tissue permeability, the volume distribution could be increased in patients with low serum albumin.

Despite the wealth of published data relating linezolid exposure values with toxicity (Table 1), among the published reports, the toxicity thresholds vary widely. What is more, the analyzed toxicity was mainly focused on the thrombocytopenia. Although thrombocytopenia is the most notable example of dose-dependent toxicity with linezolid, it should not be overlooked that hyperlactacidemia is another dose-dependent toxic effect occurring during linezolid treatment, which sometimes may be life-threatening (Pea et al., 2006). In the current study, thrombocytopenia, anemia, leukopenia, and hyperlactacidemia were observed in the included patients. However, other linezolid-related toxicity such as gastrointestinal intolerance, allergic skin rash, and visual disturbances were not included because those were not routinely recorded in the medical record system.

Using a validated Bayesian approach to estimate AUC or trough concentration of linezolid and CART analysis, thresholds in AUC and C_min_ over the first 48 h of linezolid treatment and steady state that impacted toxicity risk were derived. The CART-derived thresholds are inherently able to provide linezolid exposure thresholds, where the incidence of linezolid toxicity is most disproportionate. To compare the CART-derived thresholds, we assessed the predictive performance of these thresholds and other possible linezolid-related toxicity. The negative and positive predictive values of the CART-derived thresholds were high. According to the area under the ROC curve, the value of the AUC_0-24_ and C_min_ thresholds at day 2 and steady state were close and higher than 0.79. Therefore, the C_min_ threshold at day 2 and steady state of ≥6.9 mg/L could be the optimal choice because it is easier to implement in clinical practice without obtaining multiple samples or using Bayesian software tools to estimate AUC_0-24_. The C_min_ threshold of ≥6.9 mg/L was close to the threshold reported by Pea et al. (2012). In addition, we also compared the exposure at initial and steady states in determining linezolid-related toxicity. Compared with the AUC_0-24_ threshold at day 2 and steady state, the area under the ROC curve of threshold of AUC_0-24, d1_ ≥163 mg h/L was lower, and the negative predictive value was higher, but the positive predictive value was lower. Even so, the value of the area under the ROC curve was >0.75 and the positive predictive value was 64.04%, which indicated that the threshold of AUC_0-24, d1_ ≥163 mg h/L was of potential to capture linezolid exposure that preceded the toxicity at initial therapy. However, it was pity that no threshold was discovered for C_min_ at the first 24 h after initial linezolid treatment, which limits the popularization of monitoring the initial exposure in clinic as AUC estimation is cumbersome. Fortunately, the predictive performance of C_min_ at day 2 and steady state was close, indicating the initial monitoring of C_min_ at day 2 has the potential to predict linezolid-related toxicity, which is of great significance for patients with early-onset toxicity. Furthermore, we noted that the C_min_ threshold at day 2 and steady state was the same. It is because the t_1/2_ of linezolid is 3.4–7.4 h, which means for most patients, it could achieve steady state after treatment of 48 h. In this study, we define the steady state as patients who received at least five doses of linezolid, especially for those patients who received linezolid every 24 h.

It was notable in the final logistic regression models that the duration of linezolid is another independent risk factor for linezolid-related toxicity, besides the exposure thresholds. The Kaplan–Meier plot revealed that the median time from the initiation of therapy to the development of myelosuppression was 12 days, which was close to that reported by others (Tsuji et al., 2017). In addition, CrCL was included as the independent risk factor when the exposure variable was AUC_0-24, d1_ ≥163 mg h/L. It might be that the AUC over the first 24 h after initial linezolid treatment was not enough to reflect the renal function, but with the increased times of administration, the exposure differs widely between patients with various renal functions.

There are some limitations in our study as follows. First, the retrospective nature of the study and the thresholds attained based on the predictive model might limit the applicability of some of our conclusions. Second, the final model of linezolid was only internally validated, and external validation was not implemented. Third, the CART-derived thresholds for linezolid-related toxicity should be confirmed prospectively. The linezolid toxicities analyzed in the current study did not include all of the linezolid-associated side effects.

In conclusion, the present study derived linezolid exposure–toxicity thresholds in AUC and C_min_ over the first 48 h of linezolid treatment and steady state. The predictive performance of CART-derived C_min_ thresholds at 48 h and steady state were comparable to that of AUC_0-24_ thresholds. Considering that the AUC estimation is cumbersome, the C_min_ threshold at 48 h and steady state with a value of ≥6.9 mg/L is recommended in clinical practice to guide dosage adjustment, especially in patients with renal insufficiency and patients with low serum albumin.

## Data Availability

The raw data supporting the conclusions of this article will be made available by the authors, without undue reservation.
