# Supplementary material for: Model Based Identification of Linezolid Exposure–toxicity Thresholds in Hospitalized Patients
Source: Front Pharmacol. 2021 Oct 5;12:732503. doi: 10.3389/fphar.2021.732503 (PMC8525462; doi:10.3389/fphar.2021.732503)
Supplement: Supplementary file 1 [file DataSheet1.docx]

**Table S1 Population pharmacokinetic parameter estimates from the base model**

| **Parameter** | **Estimate** | **RSE** | **shrinkage** |
| --- | --- | --- | --- |
| **Fixed Effects** |  |  |  |
| TVF | 0.734 | 12% |  |
| TVKA[h^-1^] | 1.04 | 27% |  |
| TVCL[L/h] | 6.04 | 8% |  |
| TVV[L] | 53.6 | 13% |  |
| **Between-subject Variability (BSV)** |  |  |  |
| BSV_CL [%CV] | 41.10% | 7% | 6% |
| BSV_V [%CV] | 25.90% | 34% | 32% |
| **Residual Variability (RV)** |  |  |  |
| Proportional Error [%CV] | 20% | 26% | 31% |

a. BSV calculated as $\sqrt{e^{\omega^{2}}-1}$

**Table S2 Forward selection of covariates**

| **PAR** | **COV** | **Functional Form** | **DF** | **Min,ROUND, &COVAR Successful** | **OFV** | **AIC** | **BSC_CL** | **BSV_V** |
| --- | --- | --- | --- | --- | --- | --- | --- | --- |
| **Round 1 (Reference Model: Base)** | | | | | | | | |
| **REF** | **--** | **--** | **--** | **--** | **673.602** | **687.602** | **41.10%** | **25.90%** |
| CL | SEX | Add Shift | 1 | YES | 673.589 | 689.589 | 41% | 26% |
| CL | AGE | Linear | 1 | YES | 658.89 | 674.89 | 39.20% | 25.20% |
| CL | AGE | Allometric | 1 | YES | 658.861 | 674.861 | 39% | 25.20% |
| CL | AGE | Power | 1 | YES | 660.07 | 676.07 | 39.40% | 25.50% |
| CL | AGE | Exponent | 1 | YES | 660.07 | 676.07 | 39.40% | 25.50% |
| CL | WT | Linear | 1 | YES | 671.983 | 687.983 | 40.90% | 25.90% |
| CL | WT | Allometric | 1 | YES | 676.945 | 692.945 | 41.40% | 1.10% |
| CL | WT | Power | 1 | YES | 671.754 | 687.754 | 40.90% | 25.90% |
| CL | WT | Exponent | 1 | YES | 671.754 | 687.754 | 40.90% | 25.90% |
| CL | HT | Linear | 1 | YES | 672.005 | 688.005 | 40.90% | 25.90% |
| CL | HT | Allometric | 1 | YES | 672.006 | 688.006 | 40.90% | 25.90% |
| CL | HT | Power | 1 | YES | 671.897 | 687.897 | 40.90% | 25.90% |
| CL | HT | Exponent | 1 | YES | 671.898 | 687.898 | 40.90% | 25.90% |
| CL | WBC | Linear | 1 | NO | 1121.213 | 1137.213 | 31.20% | 31.20% |
| CL | WBC | Allometric | 1 | NO | -- | -- | -- | -- |
| CL | WBC | Power | 1 | YES | 670.463 | 686.463 | 40.50% | 26.40% |
| CL | WBC | Exponent | 1 | NO | -- | -- | -- | -- |
| CL | ALB | Linear | 1 | YES | 663.688 | 679.688 | 41.40% | 27.80% |
| CL | ALB | Allometric | 1 | YES | 663.688 | 679.688 | 41.40% | 27.80% |
| CL | ALB | Power | 1 | YES | 663.197 | 679.197 | 41.20% | 27.70% |
| CL | ALB | Exponent | 1 | YES | 663.197 | 679.197 | 41.20% | 27.70% |
| **CL** | **CRCL** | **Linear** | **1** | **YES** | **608.326** | **624.326** | **34.20%** | **28.90%** |
| CL | CRCL | Allometric | 1 | YES | 608.326 | 624.326 | 34.20% | 28.90% |
| CL | CRCL | Power | 1 | YES | 611.744 | 627.744 | 34.80% | 26.60% |
| CL | CRCL | Exponent | 1 | YES | 611.744 | 627.744 | 34.80% | 26.60% |
| V | SEX | Add Shift | 1 | YES | 673.502 | 689.502 | 41.20% | 25.70% |
| V | AGE | Linear | 1 | YES | 672.719 | 688.719 | 40.50% | 26.10% |
| V | AGE | Allometric | 1 | YES | 672.719 | 688.719 | 40.50% | 26.10% |
| V | AGE | Power | 1 | YES | 672.494 | 688.494 | 40.40% | 26.10% |
| V | AGE | Exponent | 1 | YES | 672.494 | 688.494 | 40.40% | 26.10% |
| V | WT | Linear | 1 | YES | 673.239 | 689.239 | 41.00% | 26.10% |
| V | WT | Allometric | 1 | YES | 673.239 | 689.239 | 41.00% | 26.10% |
| V | WT | Power | 1 | YES | 673.291 | 689.291 | 41.10% | 26.10% |
| V | WT | Exponent | 1 | YES | 673.291 | 689.291 | 41.10% | 26.10% |
| V | HT | Linear | 1 | YES | 673.573 | 689.573 | 41.10% | 26.00% |
| V | HT | Allometric | 1 | YES | 673.573 | 689.573 | 41.10% | 26.00% |
| V | HT | Power | 1 | YES | 673.576 | 689.576 | 41.10% | 26.00% |
| V | HT | Exponent | 1 | YES | 673.576 | 689.576 | 41.10% | 26.00% |
| V | WBC | Linear | 1 | YES | 672.786 | 688.786 | 41.60% | 25.00% |
| V | WBC | Allometric | 1 | YES | 672.786 | 688.786 | 41.60% | 25.00% |
| V | WBC | Power | 1 | YES | 672.924 | 688.924 | 41.50% | 25.20% |
| V | WBC | Exponent | 1 | YES | 672.924 | 688.924 | 41.50% | 25.20% |
| V | ALB | Linear | 1 | YES | 662.939 | 678.939 | 41.00% | 27.60% |
| V | ALB | Allometric | 1 | YES | 662.968 | 678.968 | 41.10% | 27.70% |
| V | ALB | Power | 1 | YES | 663.334 | 679.334 | 40.90% | 27.70% |
| V | ALB | Exponent | 1 | YES | 663.334 | 679.334 | 40.90% | 27.70% |
| V | CRCL | Linear | 1 | YES | 657.795 | 673.795 | 38.30% | 17.80% |
| V | CRCL | Allometric | 1 | YES | 657.796 | 673.796 | 38.30% | 17.80% |
| V | CRCL | Power | 1 | NO | -- | -- | -- | -- |
| V | CRCL | Exponent | 1 | YES | 658.464 | 674.464 | 38.20% | 20.50% |
| **Round 2 (Reference Model=Base+CRCL-CL)** | | | | | | | | |
| **REF** | **--** | **--** | **--** | **--** | **608.326** | **624.326** | **34.20%** | **28.90%** |
| CL | SEX | Add Shift | 1 | YES | 607.647 | 625.647 | 34.20% | 28.50% |
| CL | AGE | Linear | 1 | YES | 605.408 | 623.408 | 34.10% | 28.10% |
| CL | AGE | Allometric | 1 | YES | 605.45 | 623.45 | 34.10% | 28.40% |
| CL | AGE | Power | 1 | YES | 605.65 | 623.65 | 34.10% | 28.50% |
| CL | AGE | Exponent | 1 | YES | 605.65 | 623.65 | 34.10% | 28.50% |
| CL | WT | Linear | 1 | YES | 605.369 | 623.369 | 33.90% | 28.60% |
| CL | WT | Allometric | 1 | YES | 606.682 | 624.682 | 34.10% | 28.90% |
| CL | WT | Power | 1 | YES | 606.638 | 624.638 | 34.10% | 28.90% |
| CL | WT | Exponent | 1 | YES | 606.638 | 624.638 | 34.10% | 28.90% |
| CL | HT | Linear | 1 | YES | 606.155 | 624.155 | 33.90% | 28.60% |
| CL | HT | Allometric | 1 | YES | 606.169 | 624.169 | 33.90% | 28.60% |
| CL | HT | Power | 1 | YES | 606.099 | 624.099 | 33.90% | 28.60% |
| CL | HT | Exponent | 1 | YES | 606.098 | 624.098 | 33.90% | 28.60% |
| CL | WBC | Linear | 1 | YES | 600.009 | 618.009 | 32.70% | 29.50% |
| CL | WBC | Allometric | 1 | YES | 601.491 | 619.491 | 32.70% | 31.50% |
| CL | WBC | Power | 1 | YES | 601.269 | 619.269 | 32.90% | 29.80% |
| CL | WBC | Exponent | 1 | NO | -- | -- | -- | -- |
| CL | ALB | Linear | 1 | YES | 597.028 | 615.028 | 34.40% | 31.40% |
| CL | ALB | Allometric | 1 | YES | 595.804 | 614.804 | 34.40% | 32.10% |
| CL | ALB | Power | 1 | YES | 595.079 | 614.079 | 34.20% | 32.10% |
| CL | ALB | Exponent | 1 | YES | 595.079 | 614.079 | 34.20% | 32.20% |
| V | SEX | Add Shift | 1 | YES | 607.751 | 625.751 | 34.10% | 28.80% |
| V | AGE | Linear | 1 | YES | 608.319 | 626.319 | 34.20% | 28.80% |
| V | AGE | Allometric | 1 | YES | 608.319 | 626.319 | 34.20% | 28.80% |
| V | AGE | Power | 1 | YES | 608.317 | 626.317 | 34.20% | 28.80% |
| V | AGE | Exponent | 1 | YES | 608.317 | 626.317 | 34.20% | 28.80% |
| V | WT | Linear | 1 | YES | 608.157 | 626.157 | 34.20% | 28.80% |
| V | WT | Allometric | 1 | YES | 608.157 | 626.157 | 34.20% | 28.80% |
| V | WT | Power | 1 | YES | 608.145 | 626.145 | 34.20% | 28.80% |
| V | WT | Exponent | 1 | YES | 608.145 | 626.145 | 34.20% | 28.80% |
| V | HT | Linear | 1 | YES | 607.963 | 625.963 | 34.20% | 29.10% |
| V | HT | Allometric | 1 | YES | 607.889 | 625.889 | 34.10% | 29.00% |
| V | HT | Power | 1 | YES | 607.913 | 625.913 | 34.10% | 28.90% |
| V | HT | Exponent | 1 | YES | 607.913 | 625.913 | 34.10% | 28.90% |
| V | WBC | Linear | 1 | YES | 608.276 | 626.276 | 34.40% | 28.60% |
| V | WBC | Allometric | 1 | YES | 608.276 | 626.276 | 34.40% | 28.60% |
| V | WBC | Power | 1 | YES | 608.286 | 626.286 | 34.40% | 28.70% |
| V | WBC | Exponent | 1 | YES | 608.286 | 626.286 | 34.40% | 28.70% |
| **V** | **ALB** | **Linear** | **1** | **YES** | **590.674** | **608.674** | **34.20%** | **32.70%** |
| V | ALB | Allometric | 1 | YES | 590.712 | 608.712 | 34.40% | 32.70% |
| V | ALB | Power | 1 | YES | 591.948 | 609.948 | 33.90% | 33.20% |
| V | ALB | Exponent | 1 | YES | 591.948 | 609.948 | 33.90% | 33.20% |
| V | CRCL | Linear | 1 | YES | 603.956 | 621.956 | 33.50% | 32.40% |
| V | CRCL | Allometric | 1 | YES | 603.956 | 621.956 | 33.50% | 32.40% |
| V | CRCL | Power | 1 | YES | 604.432 | 622.432 | 33.60% | 32.40% |
| V | CRCL | Exponent | 1 | YES | 604.432 | 622.432 | 33.60% | 32.40% |
| **Round 3 (Reference Model=Base+CRCL-CL+ALB-V)** | | | | | | | | |
| **REF** | **--** | **--** | **--** | **--** | **590.674** | **608.674** | **34.20%** | **32.70%** |
| CL | SEX | Add Shift |  |  |  |  |  |  |
| CL | AGE | Linear | 1 | YES | 619.863 | 639.863 | 37.90% | 35.20% |
| CL | AGE | Allometric | 1 | YES | 587.832 | 607.832 | 33.90% | 32.40% |
| CL | AGE | Power | 1 | YES | 587.965 | 607.965 | 33.90% | 32.60% |
| CL | AGE | Exponent | 1 | YES | 587.965 | 607.965 | 33.90% | 32.60% |
| CL | WT | Linear | 1 | YES | 587.838 | 607.838 | 33.90% | 32.70% |
| CL | WT | Allometric | 1 | NO | 602.928 | 622.928 | 35.40% | 0.30% |
| CL | WT | Power | 1 | YES | 589.273 | 609.273 | 34.10% | 33.00% |
| CL | WT | Exponent | 1 | YES | 589.272 | 609.272 | 34.10% | 33.00% |
| CL | HT | Linear | 1 | YES | 588.434 | 608.434 | 33.90% | 32.40% |
| CL | HT | Allometric | 1 | YES | 588.336 | 608.336 | 34.10% | 32.60% |
| CL | HT | Power | 1 | YES | 588.254 | 608.254 | 34.10% | 32.60% |
| CL | HT | Exponent | 1 | YES | 588.254 | 608.254 | 34.10% | 32.60% |
| **CL** | **WBC** | **Linear** | **1** | **YES** | **578.559** | **598.559** | **32.40%** | **33.60%** |
| CL | WBC | Allometric | 1 | YES | 580.625 | 600.625 | 32.90% | 31.60% |
| CL | WBC | Power | 1 | YES | 580.279 | 600.279 | 32.60% | 33.80% |
| CL | WBC | Exponent | 1 | NO | -- | -- | -- | -- |
| CL | ALB | Linear | 1 | NO | 629.775 | 649.775 | 39.10% | 0.30% |
| CL | ALB | Allometric | 1 | NO | 603.878 | 623.878 | 35.60% | 0.30% |
| CL | ALB | Power | 1 | YES | 590.312 | 610.312 | 34.20% | 32.70% |
| CL | ALB | Exponent | 1 | YES | 590.312 | 610.312 | 34.20% | 32.70% |
| V | SEX | Add Shift | 1 | YES | 590.348 | 610.348 | 34.10% | 32.70% |
| V | AGE | Linear | 1 | YES | 590.221 | 610.221 | 34.20% | 32.70% |
| V | AGE | Allometric | 1 | YES | 590.508 | 610.508 | 34.20% | 32.70% |
| V | AGE | Power | 1 | YES | 590.498 | 610.498 | 34.10% | 32.70% |
| V | AGE | Exponent | 1 | YES | 590.498 | 610.498 | 34.10% | 32.70% |
| V | WT | Linear | 1 | YES | 590.674 | 610.674 | 34.20% | 32.70% |
| V | WT | Allometric | 1 | YES | 590.677 | 610.677 | 34.20% | 32.70% |
| V | WT | Power | 1 | YES | 590.674 | 610.674 | 34.20% | 32.70% |
| V | WT | Exponent | 1 | YES | 590.674 | 610.674 | 34.20% | 32.70% |
| V | HT | Linear | 1 | YES | 590.645 | 610.645 | 34.10% | 32.90% |
| V | HT | Allometric | 1 | YES | 590.646 | 610.646 | 34.20% | 32.90% |
| V | HT | Power | 1 | YES | 590.646 | 610.646 | 34.20% | 32.90% |
| V | HT | Exponent | 1 | YES | 590.646 | 610.646 | 34.20% | 32.90% |
| V | WBC | Linear | 1 | YES | 590.617 | 610.617 | 34.10% | 33.00% |
| V | WBC | Allometric | 1 | YES | 590.448 | 610.448 | 33.90% | 33.30% |
| V | WBC | Power | 1 | YES | 590.476 | 610.476 | 33.90% | 33.20% |
| V | WBC | Exponent | 1 | YES | 590.475 | 610.475 | 33.90% | 33.20% |
| V | CRCL | Linear | 1 | YES | 590.617 | 610.617 | 34.10% | 33.00% |
| V | CRCL | Allometric | 1 | YES | 585.884 | 605.884 | 33.50% | 34.80% |
| V | CRCL | Power | 1 | YES | 586.457 | 606.457 | 33.60% | 34.90% |
| V | CRCL | Exponent | 1 | YES | 586.457 | 606.457 | 33.60% | 34.90% |
| **Round 4 (Reference Model=Base+CRCL-CL+ALB-V+WBC-CL)** | | | | | | | | |
| **REF** | **--** | **--** | **--** | **--** | **578.559** | **598.559** | **32.40%** | **33.60%** |
| CL | SEX | Add Shift | 1 | YES | 577.863 | 599.863 | 32.40% | 31.60% |
| CL | AGE | Linear | 1 | YES | 575.7 | 597.7 | 32.10% | 33.00% |
| CL | AGE | Allometric | 1 | YES | 575.225 | 597.225 | 32.10% | 33.50% |
| CL | AGE | Power | 1 | YES | 575.367 | 597.367 | 32.20% | 33.50% |
| CL | AGE | Exponent | 1 | NO | 575.349 | 597.349 | 32.10% | 33.50% |
| CL | WT | Linear | 1 | NO | -- | -- | -- | -- |
| CL | WT | Allometric | 1 | YES | 576.511 | 598.511 | 32.10% | 33.80% |
| CL | WT | Power | 1 | YES | 576.433 | 598.433 | 32.10% | 33.90% |
| CL | WT | Exponent | 1 | YES | 576.413 | 598.413 | 32.10% | 33.90% |
| CL | HT | Linear | 1 | YES | 576.455 | 598.455 | 32.20% | 33.30% |
| CL | HT | Allometric | 1 | YES | 575.719 | 597.719 | 32.10% | 33.30% |
| CL | HT | Power | 1 | YES | 576.433 | 598.433 | 32.10% | 33.90% |
| CL | HT | Exponent | 1 | YES | 575.596 | 597.596 | 32.10% | 33.30% |
| CL | ALB | Linear | 1 | YES | 577.47 | 599.47 | 32.60% | 33.50% |
| CL | ALB | Allometric | 1 | YES | 577.803 | 599.803 | 32.60% | 33.80% |
| CL | ALB | Power | 1 | NO | 592.759 | 614.759 | 34.20% | 0.30% |
| CL | ALB | Exponent | 1 | YES | 577.776 | 599.776 | 32.60% | 33.80% |
| V | SEX | Add Shift | 1 | NO | 578.086 | 600.086 | 32.20% | 33.60% |
| V | AGE | Linear | 1 | YES | 578.137 | 600.137 | 32.40% | 33.60% |
| V | AGE | Allometric | 1 | YES | 578.405 | 600.405 | 32.40% | 33.60% |
| V | AGE | Power | 1 | YES | 578.398 | 600.398 | 32.20% | 33.60% |
| V | AGE | Exponent | 1 | YES | 578.398 | 600.398 | 32.40% | 33.60% |
| V | WT | Linear | 1 | NO | 578.412 | 600.412 | 32.20% | 33.60% |
| V | WT | Allometric | 1 | YES | 578.489 | 600.489 | 32.20% | 33.60% |
| V | WT | Power | 1 | YES | 578.49 | 600.49 | 32.20% | 33.60% |
| V | WT | Exponent | 1 | YES | 578.49 | 600.49 | 32.20% | 33.60% |
| V | HT | Linear | 1 | NO | 578.483 | 600.483 | 32.20% | 33.80% |
| V | HT | Allometric | 1 | NO | 578.51 | 600.51 | 32.40% | 33.80% |
| V | HT | Power | 1 | NO | 578.51 | 600.51 | 32.40% | 33.80% |
| V | HT | Exponent | 1 | NO | 578.51 | 600.51 | 32.40% | 33.80% |
| **V** | **WBC** | **Linear** | **1** | **YES** | **570.682** | **592.682** | **32.40%** | **31.10%** |
| V | WBC | Allometric | 1 | YES | 572 | 594 | 32.40% | 31.40% |
| V | WBC | Power | 1 | NO | 572.851 | 594.851 | 32.40% | 31.80% |
| V | WBC | Exponent | 1 | YES | 572.849 | 594.849 | 32.40% | 31.80% |
| V | CRCL | Linear | 1 | YES | 574.422 | 595.422 | 32.40% | 34.80% |
| V | CRCL | Allometric | 1 | YES | 572.496 | 594.496 | 31.60% | 35.60% |
| V | CRCL | Power | 1 | YES | 573.265 | 595.265 | 31.80% | 35.80% |
| V | CRCL | Exponent | 1 | YES | 573.265 | 595.265 | 31.80% | 35.80% |
| **Round 5 (Reference Model=Base+CRCL-CL+ALB-V+WBC-CL+WBC-V)** | | | | | | | | |
| **REF** | **--** | **--** | **--** | **--** | **570.682** | **592.682** | **32.40%** | **31.10%** |
| CL | SEX | Add Shift | 1 | YES | 570.079 | 594.079 | 32.40% | 30.90% |
| CL | AGE | Linear | 1 | YES | 567.461 | 591.461 | 32.20% | 31.00% |
| CL | AGE | Allometric | 1 | YES | 568.153 | 592.153 | 32.20% | 30.60% |
| CL | AGE | Power | 1 | YES | 567.609 | 591.609 | 32.20% | 31.10% |
| CL | AGE | Exponent | 1 | YES | 567.609 | 591.609 | 32.20% | 31.10% |
| CL | WT | Linear | 1 | YES | 567.624 | 591.624 | 32.10% | 31.20% |
| CL | WT | Allometric | 1 | YES | 569.083 | 593.083 | 32.20% | 31.30% |
| CL | WT | Power | 1 | YES | 569.012 | 593.012 | 32.20% | 31.30% |
| CL | WT | Exponent | 1 | YES | 569.012 | 593.012 | 32.20% | 31.30% |
| CL | HT | Linear | 1 | YES | 569.126 | 593.126 | 32.20% | 31.00% |
| CL | HT | Allometric | 1 | YES | 568.333 | 592.333 | 32.10% | 31.10% |
| CL | HT | Power | 1 | YES | 568.24 | 592.24 | 32.10% | 31.10% |
| CL | HT | Exponent | 1 | YES | 568.24 | 592.24 | 32.10% | 31.10% |
| CL | ALB | Linear | 1 | YES | 570.245 | 594.245 | 32.60% | 30.90% |
| CL | ALB | Allometric | 1 | YES | 570.49 | 594.49 | 32.60% | 31.10% |
| CL | ALB | Power | 1 | YES | 570.486 | 594.486 | 32.60% | 31.10% |
| CL | ALB | Exponent | 1 | YES | 570.486 | 594.486 | 32.60% | 31.10% |
| V | SEX | Add Shift | 1 | YES | 570.317 | 594.317 | 32.20% | 31.20% |
| V | AGE | Linear | 1 | YES | 569.523 | 593.523 | 32.70% | 30.60% |
| V | AGE | Allometric | 1 | YES | 570.046 | 594.046 | 32.70% | 30.70% |
| V | AGE | Power | 1 | NO | 569.882 | 593.882 | 32.70% | 30.50% |
| V | AGE | Exponent | 1 | NO | 569.882 | 593.882 | 32.70% | 30.50% |
| V | WT | Linear | 1 | YES | 570.679 | 594.679 | 32.40% | 31.10% |
| V | WT | Allometric | 1 | YES | 570.682 | 594.682 | 32.40% | 31.10% |
| V | WT | Power | 1 | YES | 570.682 | 594.682 | 32.40% | 31.10% |
| V | WT | Exponent | 1 | YES | 570.682 | 594.682 | 32.40% | 31.10% |
| V | HT | Linear | 1 | YES | 570.58 | 594.58 | 32.40% | 31.20% |
| V | HT | Allometric | 1 | YES | 570.682 | 594.682 | 32.40% | 31.10% |
| V | HT | Power | 1 | NO | 570.518 | 594.518 | 32.40% | 31.20% |
| V | HT | Exponent | 1 | YES | 570.518 | 594.518 | 32.40% | 31.20% |
| V | CRCL | Linear | 1 | NO | 566.324 | 590.324 | 31.90% | 32.40% |
| V | CRCL | Allometric | 1 | NO | 565.969 | 589.969 | 31.80% | 33.30% |
| V | CRCL | Power | 1 | NO | 566.531 | 590.531 | 31.90% | 33.30% |
| V | CRCL | Exponent | 1 | NO | 566.531 | 590.531 | 31.90% | 33.30% |

**Table S3 Backward Elimination of covariates**

| **PAR** | **COV** | **Functional Form** | **DF** | **Min,ROUND, &COVAR Successful** | **OFV** | **AIC** | **BSC_CL** | **BSV_V** |
| --- | --- | --- | --- | --- | --- | --- | --- | --- |
| **Round 1 (Reference Model=Base+CRCL-CL+ALB-V+WBC-CL+WBC-V)** | | | | | | | | |
| **REF** | **--** | **--** | **--** | **--** | **570.682** | **592.682** | **32.40%** | **31.10%** |
| CL | CRCL | Linear | 1 | YES | 652.573 | 672.573 | 39.90% | 26.40% |
| V | ALB | Linear | 1 | YES | 591.02 | 611.02 | 32.90% | 27.00% |
| CL | WBC | Linear | 1 | YES | 586.386 | 608.386 | 33.30% | 34.80% |
| **V** | **WBC** | **Linear** | **1** | **YES** | **578.537** | **598.537** | **32.40%** | **33.60%** |
| **Round 2 (Reference Model=Base+CRCL-CL+ALB-V+WBC-CL)** | | | | | | | | |
| **REF** | **--** | **--** | **--** | **--** | **578.537** | **598.537** | **32.40%** | **33.60%** |
| CL | CRCL | Linear | 1 | YES | 658.24 | 676.24 | 40.10% | 28.30% |
| V | ALB | Linear | 1 | YES | 600.009 | 618.009 | 32.70% | 29.50% |
| CL | WBC | Linear | 1 | YES | 590.674 | 608.674 | 34.20% | 32.70% |

**Table S4 Linezolid PopPK parameter estimates from the final model and bootstrap results**

| **Parameter** | | **Final model** | | | | **shrinkage** | | | | | | **Bias (%)** | |
| --- | --- | --- | --- | --- | --- | --- | --- | --- | --- | --- | --- | --- | --- |
|  |  | **Estimate** | | **RSE(%)** | | **2.5th percentile** | | **Median Estimate** | | **97.5^th^ percentile** | |  |  |
| θ_F_ | | 0.731 | | 9 | | 0.610 | | 0.798 | | 0.932 | | -0.993 | |
| θ_KA_[h^-1^] | | 0.87 | | 23 | | 0.409 | | 0.717 | | 1.098 | | -4.78 | |
| θ_CL_[L/h] | | 3.07 | | 12 | | 2.23 | | 2.89 | | 3.63 | | -1.36 | |
| θ_V_[L] | | 93.1 | | 15 | | 66.12 | | 93.66 | | 128.9 | | -3.94 | |
| θ_1_ | | 2.37 | | 15 | | 1.56 | | 2.42 | | 3.10 | | 3.86 | |
| θ_2_ | | 0.559 | | 23 | | 0.314 | | 0.689 | | 1.06 | | 0.584 | |
| θ_3_ | | -1.33 | | 26 | | -2.20 | | -1.36 | | -0.644 | | -4.23 | |
| I**nter-individual variability** | |  | |  | |  | |  | |  | |  | |
| ω_CL_ [%] | | 32.40 | | 8 | | 27.29 | | 31.17 | | 36.77 | | -3.80 | |
| ω_V_ [%] | | 33.60 | | 24 | | 13.70 | | 22.20 | | 35.62 | | -5.93 | |
| Residual Variability | |  | |  | |  | |  | |  | |  | |
| σ_pro_(%) | | 16.09 | | 14 | | 13.62 | | 15.73 | | 18.43 | | -2.84 | |

PopPK, population pharmacokinetic; RSE(%), relative standard error; θ_F_, typical value of Bioavailability; θ_KA_, typical value of absorption rate constant; θ_CL_, typical value of apparent clearance; θ_V_, typical value of apparent volume of distribution; θ_1_, allometric value for CrCL as covariate for CL; θ_2_, allometric value for WBC as covariate for CL; θ_3_, allometric value for ALB as covariate for V; ω_CL_, square root of inter-individual variance for CL; ω_V_, square root of inter-individual variance for V; σ_pro_, residual variability for proportional error.

Bias (%)=(Median Estimate _Bootstrap_ - Estimate _Final model_)/Estimate _Final model_×100%

**Figure S1**


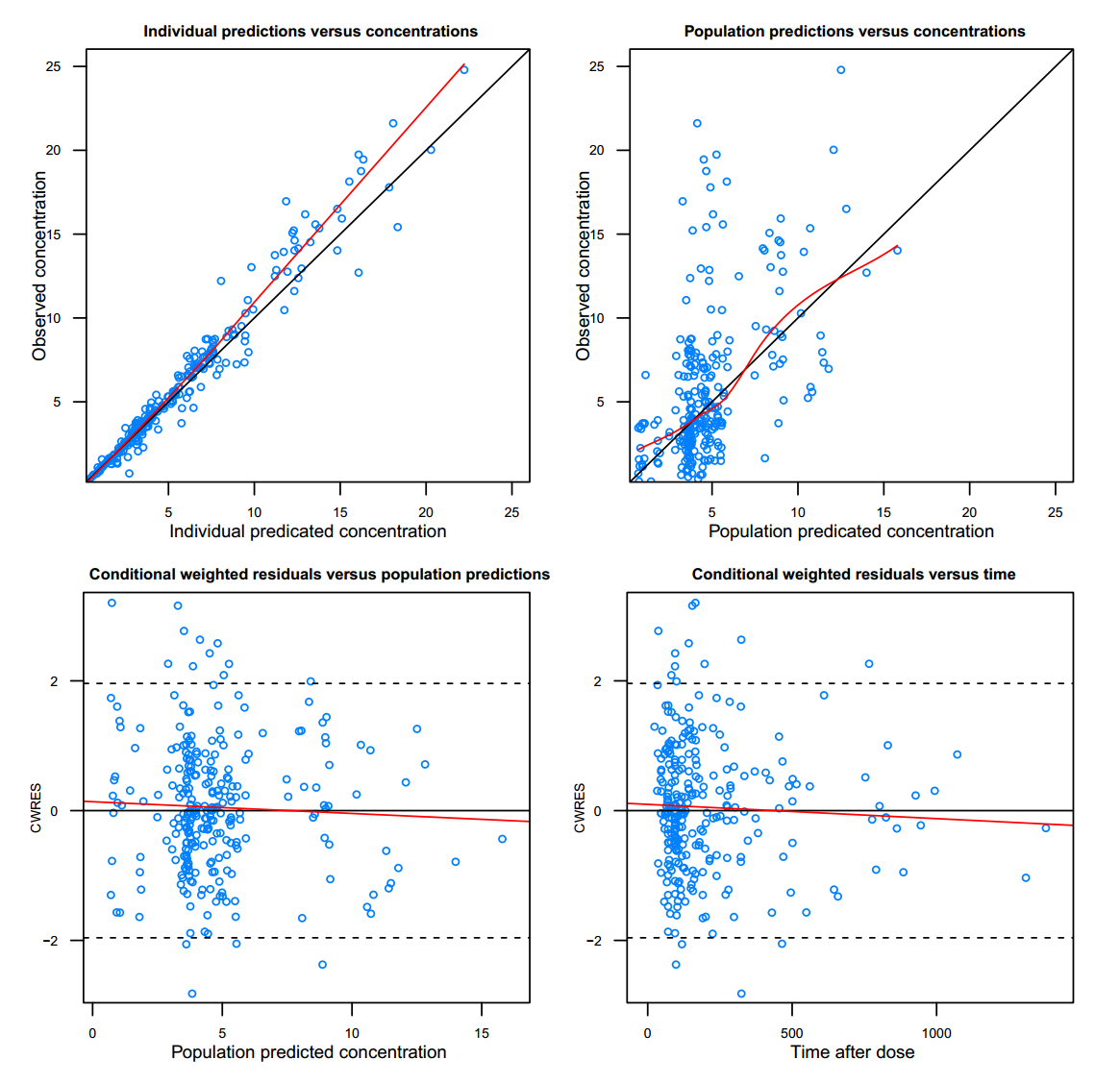


Figure S1. Diagnostic goodness-of-fit plots of the base model. a Observed concentration (DV) vs. individual predicted concentration (IPRED); b DV vs. population predicted concentration (PRED); c conditional weighted residuals (CWRES) vs. PRED; and d CWRES vs. time. The red lines in the upper panel represent loess smooth lines and linear fit lines, respectively.

**Figure S2**


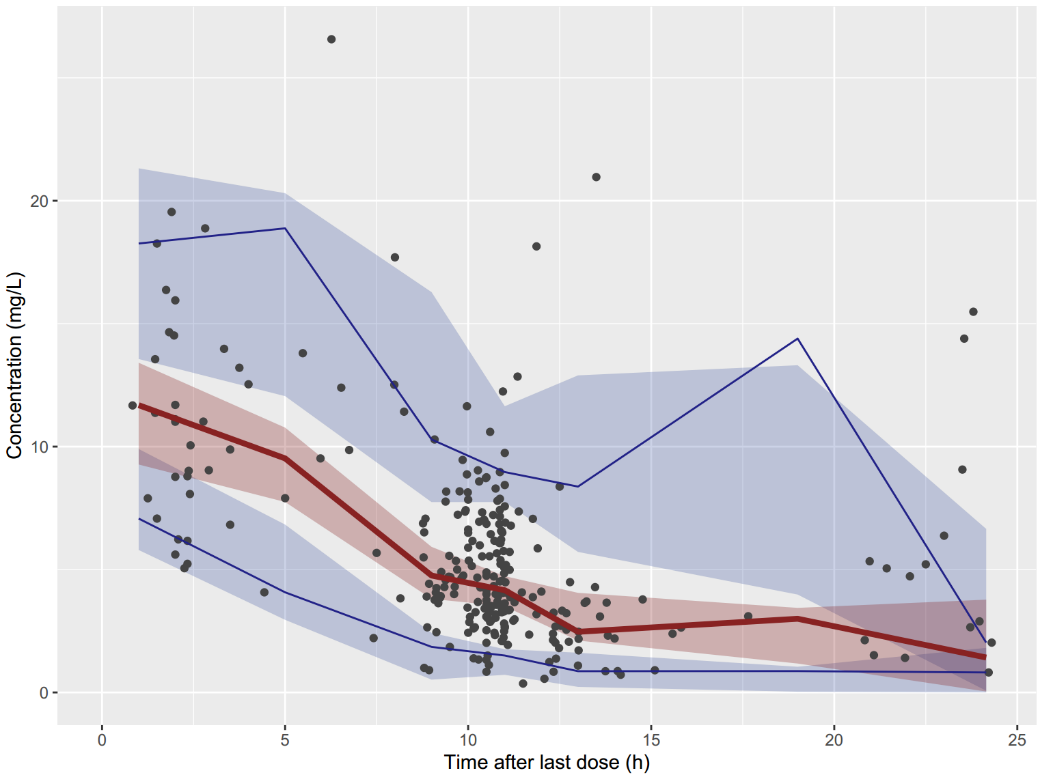


Figure S2. Prediction- and variability-corrected visual predictive check (pvcVPC) plot of the final model. The red solid lines represent the median observed concentration, and the semitransparent red fields represent the simulation-based 95% confidence intervals (CIs) for the median. The observed 5th and 95th percentiles are represented by red dashed lines, and the 95% CIs for the corresponding model predicted percentiles are shown as semitransparent blue fields. The observed concentrations are represented by dark dots.
